# Factors associated with meat hygiene-practices among meat-handlers in Metropolitan City of Kathmandu, Nepal

**DOI:** 10.1371/journal.pgph.0001181

**Published:** 2022-11-09

**Authors:** Rabindra Bhandari, Anil Kumar Singh, Prakash Raj Bhatt, Ashish Timalsina, Rama Bhandari, Pratibha Thapa, Jijeebisha Baral, Sunil Adhikari, Pramila Poudel, Sudip Chiluwal, Prakash Chandra Joshi, Nabin Adhikari

**Affiliations:** 1 Central Department of Public Health, Institute of Medicine, Kathmandu, Nepal; 2 Nepal Health Research Council, Ramshahpath, Kathmandu; 3 Faculty of Health and Allied Sciences, Pokhara University, Pokhara, Nepal; 4 Nepal Public Health and Education Group, Kathmandu, Nepal; 5 Nepal Public Health Association, Kathmandu, Nepal; 6 Research and Development Division, Dhulikhel Hospital, Dhulikhel, Nepal; Mustafa Kemal University: Hatay Mustafa Kemal Universitesi, TURKEY

## Abstract

Meat hygiene refers to all conditions and measures necessary to ensure safety and suitability of meat at all stages of the food chain. Inadequate hygiene practices allow consumers to be exposed to pathogens causing public health problems. Inadequate facilities and hygiene practices in meat shops results in meat contamination. The study aimed to identify factors associated with meat hygiene practices among meat handlers in the Metropolitan City of Kathmandu, Nepal. A cross-sectional study was designed with a semi-structured questionnaire and observation checklist that collected information about hygiene practices from 320 consenting meat-handlers by interviewer-administered technique. Data was entered in EpiData and analyzed using IBM SPSS version 21. Descriptive statistics of frequency distribution were used to report meat hygiene-practices and other independent variables, with multivariate logistic regression to establish predictors of meat hygiene-practices at 5% level of significance. The study revealed that less than half (44.4%) of the meat handlers had satisfactory meat hygiene practices. The adjusted regression analysis showed, strong evidence (p<0.01) of association of higher education level (AOR = 2.8, 95% CI = 1.7–4.5), other occupational involvement (AOR = 2.2, 95% CI = 1.9–2.4), and being officially registered (AOR = 2.8, 95% CI = 1.2–6.8) with meat hygiene practices. However, there was fair evidence (p<0.05) of association between shorter duration of meat being processed to sale (AOR = 0.57, P = 0.042) and meat hygiene practices. In this study, the satisfactory meat hygiene practices of meat handlers was low. The educational level, registration status of shops, involvement in other jobs, and awareness on meat hygiene were identified as key factors associated with meat hygiene practices. Thus, these factors need to be considered while developing programs to improve meat hygiene practices among the meat handlers. Meat handlers should be provided with training and orientation program for improving the meat hygiene practices.

## Introduction

Food is a basic nutrient source and hygienic food is required for the proper growth and development as well as good functioning of the body [1]. Food hygiene is a condition and measures necessary to ensure the safety of food from production to consumption. Meat products are highly perishable foods and are consumed food items on a daily basis by a large number of the population. Meat has been recognized as the chief vehicle for significant food borne illnesses [2]. Meat has appropriate moisture and nutrients which is favorable for growth of microorganisms which can cause food poisoning and zoonosis like trichinellosis, taeniasis, echinococcosis- hydatidosis [3]. Meat hygiene refers to all conditions and measures necessary to ensure the safety and suitability of meat at all stages of the food chain [4].

Food borne disease commonly occurs in developing countries due to poor hygiene and safety practices, inadequate food hygiene laws, poor regulatory mechanisms, poor investment in safety equipment, and poor education of meat handlers [5]. Improper meat handling and unhygienic practices during cutting and processing leads to cross-contamination of meat and can cause meat borne bacterial outbreaks [2,6].

According to the World Health Organization (2015), almost 1 in 10 people fall ill and 420,000 die every year from eating contaminated food [7]. After the African Region, The WHO South-East Asia Region has the second highest burden of foodborne diseases per population. More people living in the WHO South-East Asia Region fall ill and die from foodborne diseases every year than in any other WHO Region, with more than 150 million cases and 175 000 deaths a year. The highest burden is in children, accounting for 40% of illnesses and 30% of U-5 deaths annually in the South-East Asia Region [7]. Pregnant women are about 20 times more likely than other adults to get sick from *Listeria monocytogenes* which is typically found in raw meat, delicatessen products, including processed ready-to-eat meat products [8]. However, evidence on the burden of foodborne diseases due to poor meat handling practices is insufficient. Improving hygienic meat-handling practices is essential for the meat-handlers during meat production, distribution, storage and sales at retail shops to prevent or reduce microbial contamination [9].

Poor handling of raw meat is the primary cause for cross-contamination in developing countries. The behavior diagnostic function of considering knowledge and attitudinal dispositions play an important role in elucidating the dynamics of underpinning food-related diseases as antecedents in meat-hygiene practices among meat handlers, which are very important in providing the needed understanding in addressing emerging issues related to health and safety of consumers [10].

Though knowledge and attitude are key constructs of food handlers [11], studies have suggested other important psychological constructs which includes self-efficacy, subjective norms and intentions to perform behavior as well as the organizational factors such as food safety culture, resource adequacy and regulatory frameworks influencing safe food handling behaviors [12,13]. Adopting proper food handling behavior decreases the risk of foodborne illnesses, which is also an integral preventive behavior. The Health Belief Model (HBM) suggests that health behavior decisions are based on risk perception and behavioral evaluation. HBM can explain 60% of the variation in behavior intention of food handlers and self-efficacy is the most critical predictor of behavior while health consciousness indirectly affects the behavior intention[14]. The most applied theory in research involving food safety behavior has been the Theory of Planned Behavior (TPB), which illustrates that the behavior of the food handlers is directed by their intention to perform behavior and perceived behavioral control. Similarly, attitudes towards behavior, subjective norms and self-efficacy guides their intention to perform behavior [15].

WHO-FAO Codex Alimentarius has provided guidelines for safe and hygienic production of meat from handling at the production line to retail channel for consumption [4]. Policies and legislations regarding food safety and quality in Nepal currently consists of; The Food Act (1966) and Food Regulations (1970); Consumer Protection Act (1998) and Rules (2000); Slaughterhouse and Meat Inspection Act (1998) and Rules (2001); Directive on Export-Import Inspection and Quality Certification (2006); Regulations on Dietary Supplements (2016); and Directives on Safety and Quality of Meat Processing (2016), Drinking Water (2017), and Food Service Establishments (2017). Other legislative concerns cover licensing of businesses, including food businesses, and other more tangential issues. Institutionally, the major player in the food safety sector is the Ministry of Agricultural Development’s Department of Food Technology and Quality Control (DFTQC), which monitors food safety and quality and enforces food-related legislation. Most of the small meat entrepreneurs are unaware of what is involved with good hygiene practices that hampers safe meat production [16]. The lack of proper implementation of the existing meat inspection act and the policy directives by the authorities in the country has posed meat as a potential source of infection to the consumers [3].

Poor hygiene practices during slaughtering and marketing of meat, high prevalence of meat borne diseases, failure to enforce the animal slaughterhouse and meat inspection act, lack of infrastructure and awareness among different stakeholders are considered as major meat safety issues in Nepal [3]. Very few studies on meat hygiene in Nepal consider behavior theories as underpinning frameworks which considered appropriate public health principles to elucidate the dynamics of the problems associated with meat-hygiene practices. Therefore, this study aimed to identify factors associated with meat-hygiene practices among meat handlers in the Metropolitan City of Kathmandu, Nepal adding empirical evidence to ensure safety of the population at risk of ill-health resulting from consumption of contaminated meat products.

## Methods

### Study design and setting

The study conducted was a cross-sectional survey design to determine factors associated with meat hygiene practices among 320 consenting meat- handlers Kathmandu Metropolitan City (KMC) of Nepal, considering the prevalence of meat hygiene to be 47% from previous study conducted at Chitwan district [17], at a 5% margin of error and 5% level of significance. Data collection was done from February 26 to March 21 in 2020.

### Sampling technique

We used a systematic random sampling technique to select the participants. First of all, the registration list which was recently updated by the “*Nepal Maccha Mashu Byabasayi Sangh*” was obtained and segregated based on the wards of KMC. The total registered meat shop was 1200. The list contained the details of the meat shop owner, location and contact number. Thus, the shops were systematically selected (sampling interval; k = 4) for the study and contacted the day before they would be interviewed which made it easier to locate the shop and schedule the time for the interview. If the shop terminated or the owner refused to participate in the study immediate next shop in the list was contacted for inclusion in the study. We included meat handlers from an age group of more than 18 years. If there were more than one meat handler in the shop, we randomly selected one person by using lottery methods. We excluded the person working as a cashier, driver in the meat shop and/not involved in meat handling.

### Study variables

#### Dependent variables

The study considered meat-hygiene practices as the major outcome variable of concern with regards to the Codex Alimentarius commission, and identified relevant literatures reviewed [4,17,18]. This was assessed and measured based on cleanliness of equipment and utensils, cleanliness of setting, waste management, use of personal protective equipment and personal practices related to health and hygiene. The instrument designed to measure the dependent variable was a 37-item structured for hygiene-practice observations covering the domain mentioned above. Scoring was done by considering correct practices to have one point and wrong practice zero point hence generating an aggregate total score of 37-point rating scale. Finally, categorization of scores was adopted to facilitate interpretation of the measure obtained as “Not satisfactory if below mean score” and Satisfactory if above mean score. **Hygienic practices** Cleanliness of equipment and utensils Cleanliness of setting Waste management Use of personal protective equipmentPersonal practices related to health and hygiene **Socio-demographic characteristics** Age Sex Ethnicity Religion Experience in butchering Daily working hours **Awareness on meat hygiene Educational characteristics** Education level of the respondentTraining related to meat handling **Availability of facilities for hygiene, sanitation and safety**

#### Independent variables

Independent variables were selected based on previously published. Data analysis was conducted with IBM Statistical Product and Service Solutions (SPSS version 16) variables are broadly classified in sociodemographic factors, meat shop related factors, meat handlers related factors and awareness related factors.

Sociodemographic factors included age, ethnicity, education, religion and income of meat handlers. The meat shop related variable includes location of meat shop, types of meat, number of workers, registration status, slaughter within shop, means of transport. The meat handlers related factors included handlers experience, workload, source of learning meat handling and working hours. The awareness related variables included hearing about meat hygiene, meaning of meat hygiene, sources of meat contamination, information on health issues related to unhygienic meat, hygiene aspects to be followed by meat handlers. The level of awareness was measured by using the scoring based on the mean. The items were coded as 1 and 0 for correct response and incorrect, respectively. There were 46 points for the awareness level, in which the minimum response was 2 and maximum was 43 and the mean of the responses was found to be 25.9 ± 9.71. Thus, the awareness level on meat hygienic practices categorized as satisfactory level of awareness on meat hygiene (above mean) and not satisfactory level of awareness on meat hygiene (below mean)

### Tools and techniques for data collection

The study adopted, and modified instrument previously used in a study by Khanal, and Poudel, entitled “Factors associated with meat safety knowledge and practices among butchers of Chitwan in Nepal [17] which referenced the Codex Commission’s Code of Good Hygienic Practices for meat 2005 [4]. The semi-structured questionnaire contained socio-demographic information, characteristics of meat shop, meat handling characteristics of meat handler, awareness on meat hygiene and practices on meat hygiene and observation checklist for assessing meat hygienic practices of meat handlers. Translation to Nepali and back translation to English was done.

The pre-testing of the tools was done in 10 percent of the sample size from Budhanilkantha Municipality which consists the similar characteristics as study population. Internal consistency of the scale was assessed using ’Cronbach’s alpha (α)’ for hygienic practices which was 0.71. The consistency of the questionnaire was maintained and necessary modifications were made in the tools after pretesting. The completeness and consistency of the data was checked immediately after data collection.

### Data management and analysis

The data were edited, coded and entered in EpiData version 3.1 and data analysis was conducted with IBM Statistical Product and Services Solutions (SPSS version 16). Descriptive analysis (frequency and percentage) was used to report the dependent and independent variables. Frequency tables were used for categorical variables, while mean and standard deviation (SD) were calculated for continuous variables. Further, binary logistics regression was used to test the association between dependent and independent variables. All variables significant at 5% significance level in the univariate analysis were subjected to multivariate logistic regression analysis using the enter method. The adjusted and unadjusted odds ratio with 95% confidence intervals were reported.

### Ethical statements

Ethical approval was obtained from the Institutional Review Board of Institute of Medicine (IOM), Tribhuvan University while approval for study was also obtained from “Nepal Fish and Meat Seller’s Association”. The purpose and importance of the study was clearly explained before enrolling the participants in the study while the verbal and written consent was taken from the participants before conducting the interview. The participants selected in the study were asked to participate voluntarily and the confidentiality of the information was maintained by anonymizing the questionnaire. All the possible harms and advantages were fully explained before accepting them in the study.

## Results

### Distribution of participants by socio-demographic, meat shop and meat handlers related characteristics

The study presents the frequency distribution of participants by their socio demographic, meat shop and meat handlers related characteristics in Table 1. The mean age of the participants was 35.81+ 8.9 years. Majority (81.6%) of participants in this study were males. Similarly, a significant majority (76.6%) of the participants were adherents to the Hindu religion while more than half (55.0%) of the participants were from Janajati caste. Regarding education, about 45.6% of the participants had completed a secondary level of education. Only 6.6% participants had formal training on meat hygiene practice. Meat shops were the major source of income for the majority of the participants (80.6%). We found most (90.6%) of the meat shops were licensed where most of the meat handlers had 1–5 years of experience. Majority (76.6%) of the meat shops had transactions of 50 kg and less while more than half (55.6%) of the participants worked 6 to 10 hours on an average daily. Only about one in three (32.8%) meat shops had slaughtering facilities within the shop. Regarding the inspection from authorities, 67.8% were inspected by the government authorities *(See Table 1).*

**Table 1:** Distribution of participants by socio-demographic, meat shop and meat handlers related characteristics.

Characteristics	Number	Percentage
**Age**		
**Mean age**	**35.81 ± 8.9 years**	
18–28	84	26.3
29–38	129	40.3
39–48	78	24.4
49–58	27	8.4
59–68	2	0.6
**Sex**		
Male	261	81.6
Female	59	18.4
**Ethnicity**		
Brahmin/Chhetri	73	22.8
Janajati	176	55.0
Dalit	14	4.6
Madhesi	16	5.3
Muslim	41	12.8
**Religion**		
Hindu	245	76.6
Buddhist	26	8.1
Muslim	41	12.8
Christian	8	2.5
**Educational status**		
Not any formal education	23	7.2
Primary	128	40.0
Secondary	146	45.6
Higher education	23	7.2
**Major source of income**		
Agriculture	4	1.3
Business	43	13.4
Meat shop	258	80.6
Government service	2	0.6
Non-government service	10	3.1
Foreign service	3	0.9
**Source of learning meat handling practice**		
Parents	76	23.8
Relatives/friends/self	223	69.7
Formal training	21	6.6
**License**		
Yes	290	90.6
No	30	9.4
**Experience in meat handling in years**		
1–5	140	44
6–10	106	33.3
11–15	48	15.1
16–20	21	6.6
21–25	5	1.6
**Slaughter within shop**		
Yes	105	32.8
No	215	67.2
**If no, means of transport**		
Carrying self	10	4.7
Motorcycle	112	52.1
Car/jeep/van	93	43.3
**Average meat transaction**		
50 kg and less	245	76.6
More than 50 kg	75	23.4
**Average work load in a day**		
20 kg and less	211	65.9
More than 20 kg	109	34.1
**Involvement in other activities**		
Yes	56	17.5
No	264	82.5
**Average working hours**		
Up to 5 hours	32	10.0
6 to 10 hours	178	55.6
11 to 15 hours	110	34.4
**Inspection by authority**		
No	105	32.2
Yes	215	67.8

### Distribution of participants by availability of basic amenities based on observation

The study further showed, based on observations, that 90.6% of participants reported use of clean and rust-free knife in meat-hygiene practices with respect to availability of amenities. Similarly, the majority (75.3%) of participants reported cleaning their knives before each new period of work and the majority (67.8%) had practiced cleaning utensils after use. Majority (75%) of the participants had kept the cutting boards in a good condition. Regarding observation on the cleanliness of settings; the floor was found impervious and clean in more than half (57.8%) of the shops. Majority (92%) of the shops had adequate ventilation (area for air exchange). Majority (86.3%) had a proper drainage facility. Flies, insects and stray dogs could be seen in more than half of the surroundings of meat shops. Majority (66.6%) of shops had practice of using a meat net or placed inside the glass seal during display. Almost all the shops had used the municipal waste disposal system for waste management. Regarding the personal habits related to health and hygiene, half of the participants had practice of wiping hands with common cloths and the vast majority (85.9%) were handling money in between the meat handling time which poses risk of cross contamination. Similarly, most (83.4%) of the participants in the present study had the practice of cleaning hands where about (70.9%) of them had the habit of washing hands after the meat handling *(See Table 2).*

**Table 2:** Observations on equipment, setting and waste management practices of participants.

Characteristics	Yes n (%)	No n (%)
**Cleanliness of equipment**		
Knife (clean and rust free)	290 (90.6)	30 (9.4)
Knife cleaning before each new period of work	241 (75.3)	79 (24.7)
Cut board in good condition	240 (75)	80 (25)
Cut board cleaning before each new period of work	221 (69)	99 (31)
Weighing machine clean	253 (79)	67 (21)
Utensils clean	217 (67.8)	103 (32.2)
Utensils sanitize before each period of work	113 (35.3)	207 (64.7)
**Cleanliness of setting**		
Bottom floor impervious and clean	185 (57.8)	135 (42.2)
Walls impervious and clean	183 (57.2)	137 (42.8)
Slab in good condition	170 (53.1)	150 (46.9)
Ceiling clean	157 (49.1)	163 (50.9)
Ventilation (enough air exchange area)	295 (92)	25 (8)
Separation of clean and dirty areas	207 (64.7)	113 (35.3)
Drainage available	276 (86.3)	44 (13.8)
Presence of insects and stray animals	168 (52.5)	152 (47.5)
Meat display with covered net	213 (66.6)	107 (33.4)
**Lairage present in 40 meat shops**		
Lairage area clean	22 (55)	18 (45)
Feeds for animals clean	6 (15)	34 (85)
Clean drinking water for them	14 (35)	26 (65)
Drainage/waste flow from lairage	26 (65)	14 (35)
**Waste management**		
Frequent waste disposal in specified area	250 (78.1)	70 (21.9)
Bury in pit	10 (3.1)	310 (96.9)
Municipal waste	316 (98.8)	4 (1.2)
Fishery use	12 (3.8)	308 (96.2)
Feed dog cat and animals	63 (19.7)	357 (81.3)
**Use of PPE**		
Use Apron	253 (79.1)	67 (20.9)
If using Apron clean or not	169 (54.3)	84 (35.7)
Use gloves	42 (13.1)	278 (86.9)
If using Gloves clean or not	18 (44.2)	26 (55.8)
Water Boots	56 (17.5)	264 (82.5)
If using Boots clean or not	19 (33.9)	37 (66.1)
Use mask	77 (24.1)	243 (75.9)
If using mask clean or not	71 (92)	6 (8)
**Personal habits related to health and hygiene**		
Spitting in between	52 (16.3)	268 (83.7)
Use tobacco	99 (30.9)	221 (69.1)
Wipe hands with common clothes	162 (50.6)	158 (49.4)
Counting money in between	275 (85.9)	45 (14.1)
Doing other work in between without cleaning	59 (18.4)	261 (81.6)
Nose picking	9 (2.8)	311 (97.2)
Clean hands	267 (83.4)	53 (16.6)
**Hand sanitation**		
Before	60 (18.8)	260 (81.2)
In between as well	33 (10.3)	287 (89.7)
After	227 (70.9)	93 (29.1)

### Distribution of participants by their knowledge and practice level of meat hygiene

The result of frequency distribution of participants’ responses regarding their level of practice of meat-hygiene, showed that 44.4% of the participants demonstrated satisfactory level of meat-hygienic practices while 43.4% of the participants had a satisfactory level of awareness on meat hygiene *(See Table 3)*

**Table 3:** Distribution of participants by their level of awareness and practice on meat hygiene.

Level of practice	Number	Percentage
Not satisfactory	178	55.6
Satisfactory	142	44.4
**Level of Awareness**		
Not satisfactory	181	56.6
Satisfactory	139	43.4

### Multivariate analysis of independent variables with level of meat hygiene practice

Multivariate analysis with logistic regression to identify explanatory variable predictors of level of meat-hygiene practices showed that certain socio-demographic characteristics of respondents such as educational status, their involvement in other jobs, selling time during display and the registration status of meat shops were significantly associated with satisfactory level of meat-hygiene practices. The study also found that the odds of demonstrating satisfactory meat-hygiene practices was 2.8 times higher (AOR = 2.8; 95% CI, 1.7–4.5) among the meat-handlers having secondary and higher educational levels. It was also observed that handlers not being involved in the jobs other than meat handling, handlers from registered meat shop and the handlers with meat selling time greater than 2 hours were more likely to have satisfactory meat-hygiene practices (AOR = 2.2; 95% CI, 1.9–2.4), (AOR = 2.8; 95% CI, 1.2–6.8) and (AOR = 0.57; 95% CI, 0.3–0.9) respectively *(See Table 4).*

**Table 4:** Multivariate analysis of independent variables with level of meat hygiene practice.

Characteristics	COR	95% CI	P-value	AOR	95% CI	P-value
**Sex**						
Female	Ref					
Male	2.0	(1.1–3.7)	0.019*	1.9	(0.9–3.8)	0.056
**Education**						
No-formal/primary	Ref					
Secondary/ above	2.6	(1.7–4.2)	<0.001*	2.8	(1.7–4.5)	<0.001*
**Awareness**						
Poor	Ref					
Good	1.1	(1.5–2.5)	0.019*	1.2	(0.7–2.1)	0.393
**Average workload**						
≤ 20 kg	Ref					
> 20 kg	0.6	(0.3–0.9)	0.041*	1.2	(0.7–2.1)	0.203
**Daily working hours**						
≤ 8 hours	Ref					
> 8 hours	1.6	(1.0–2.5)	0.034*	1.3	(0.7–2.2)	0.673
**Involvement in other jobs**						
Yes	Ref					
No	2.2	(1.2–4.2)	0.010*	2.2	(1.9–2.4)	<0.018*
**Time to sell**						
≤ 2 hours	Ref					
> 2 hours	0.6	(0.3–1.0)	0.106	0.57	(0.3–0.9)	0.042*
**Registration**						
No	Ref					
Yes	2.3	(1.0–5.4)	0.045*	2.8	(1.2–6.8)	0.018*

* Statistically significant at 95% level of confidence (p < 0.05).

## Discussion

Our study aimed to assess the current situation of meat hygiene practices and identify the factors associated with meat-hygiene practices among meat handlers in the Metropolitan City of Kathmandu, Nepal, it was observed the hygiene practices including cleanliness of setting, cleanliness of equipment, meat storage facilities, personal hygiene, use of PPE during meat handling and the health behaviors of meat handlers were identified as meat hygiene practices of meat handlers. We found that more than half of meat handlers in Kathmandu had less than satisfactory level of meat hygiene practices. The use of PPE is important not only for injury prevention to meat handlers but also for preventing cross contamination and maintaining hygiene during preparation and selling. However, the majority of handlers were not using the PPE including gloves, water boots and mask but the use of Apron seems higher than the study conducted in Dharan where 51.2 percent used Personal Protective equipment, 13.8 percent used Personal Protective equipment in Nigeria [19,20].

The education level plays an important role in awareness and hygiene practice [14,21]. However, only less than half of the handlers had secondary education in our study which is comparatively low in regard to countries like Nigeria and Kenya [22,23].

On a positive note, cleaning shop daily was observed in all the study sites in Kathmandu which was better compared to city of Dharan and India as well. A study conducted in Dharan city of Nepal reflects that 51.2 percent used to clean the shop daily [24]. It was even worse in India where only 23 percent cleaned the workplace [6]. Regarding the water supply most (88%) had provision of clean water and almost all had access to municipal waste management systems for disposal of by-product in our study.

Though the Slaughterhouse and Meat Inspection Act & Regulations suggests ante and post-mortem examination of animals and regular inspection of meat enterprises by meat inspector no such mechanism has been strongly implemented till date [16]. In Isiolo and Nairobi Counties [5], 90 percent and 87 percent of operators respectively handled meat along with handling money while 86 percent of the meat handlers in present study were found counting money in between meat handling. This compromises meat hygiene providing an area for cross contamination of meat.

Handling meat without any training could enhance the risk of cross contamination. According to Adams and Moss, training of food handlers regarding the basic concepts and requirements of personal hygiene plays an integral part in ensuring safe products to the consumers [19,25]. In our study, very low proportion (7%) had formal training which is comparatively lower than similar study conducted in Dharan where 30.4 percent have got training related to hygiene practice and the trained ones had comparatively better hygiene practice [26]. So, formal training for handlers in Kathmandu Metropolitan City could be recommended for improving the meat hygiene practices. The scenario of poor knowledge and practice skills of the meat handlers may be due to illiteracy and weak meat hygiene legislation. Other studies reported similar scenarios of low level of knowledge and hygienic practices among the butchers on meat hygiene, exposing the community toward potential meat-borne illnesses [27–29]. It is inevitable that improper handling and unsafe hygiene lead to contamination of meat eventually affecting the health of consumers.

Higher educational status, involvement in other jobs, being officially registered shop and smaller duration between slaughter to sale were found to be positively associated with the meat-hygiene practices in adjusted multivariable regression model. Similar findings have been reported by the study conducted in South Africa where the level of education and three or more years of experience were seen to be associated with meat hygiene practices [24]. This is in agreement with the studies conducted in Ethiopia and Kenya noted that the highest level of knowledge was significantly associated with workers who had better education (P< 0.05) [19,22,30]. Findings reported from Chitwan, Nepal where butchers without a side job had better practice on meat hygiene which resembles findings in our study. The butchers having higher workload (per kg/per day) was found to be significantly associated with satisfactory practice and the butchers who belonged to Brahmin/Chhetri caste were more likely to have satisfactory level of practice. In Chitwan, handlers having average working hours > 8 hours a day were found to be significantly associated with satisfactory hygiene practice [17].

This study elucidated the need of emphasis on education, mandating the legal registration, regulation on shorter time between slaughter to sale to improve the meat hygiene practices in Kathmandu Valley. Regulatory bodies should consider these aspects and formulate policy and regulations to guide and monitor meat enterprises of Kathmandu to safeguard the communities from potential meat-borne illnesses.

### Strengths and limitations of the study

The strengths behind the study can be random sampling, practice level was assessed based on observations rather than only relying on interviews. Being a cross-sectional study, it does not show the causal relations, however, does demonstrate the association between socio-demographic variables, handling related factors and shop related factors of meat handlers with meat hygiene practices.

## Conclusion

Overall, the meat hygiene practice of meat handlers in Kathmandu Metropolitan City was found not satisfactory. Factors such as sex of handlers, education level, license/registration status of shop, involvement of meat handler in other job, awareness on meat hygiene practices were identified as key factors associated with meat hygiene practices. Thus, these factors need to be addressed through training, routine monitoring and supervision from authorities to improve meat hygiene practice.

## Supporting information

S1 Data (SAV)

S1 File Questionnaire. (DOCX)

S2 File Ethical approval. (DOCX)
