# Supplementary figures and images for: Factors associated with meat hygiene-practices among meat-handlers in Metropolitan City of Kathmandu, Nepal
Source: PLOS Glob Public Health. 2022 Nov 9;2(11):e0001181. doi: 10.1371/journal.pgph.0001181 (PMC10021547; doi:10.1371/journal.pgph.0001181)

**ETHICAL CLEARANCE FROM IOM**


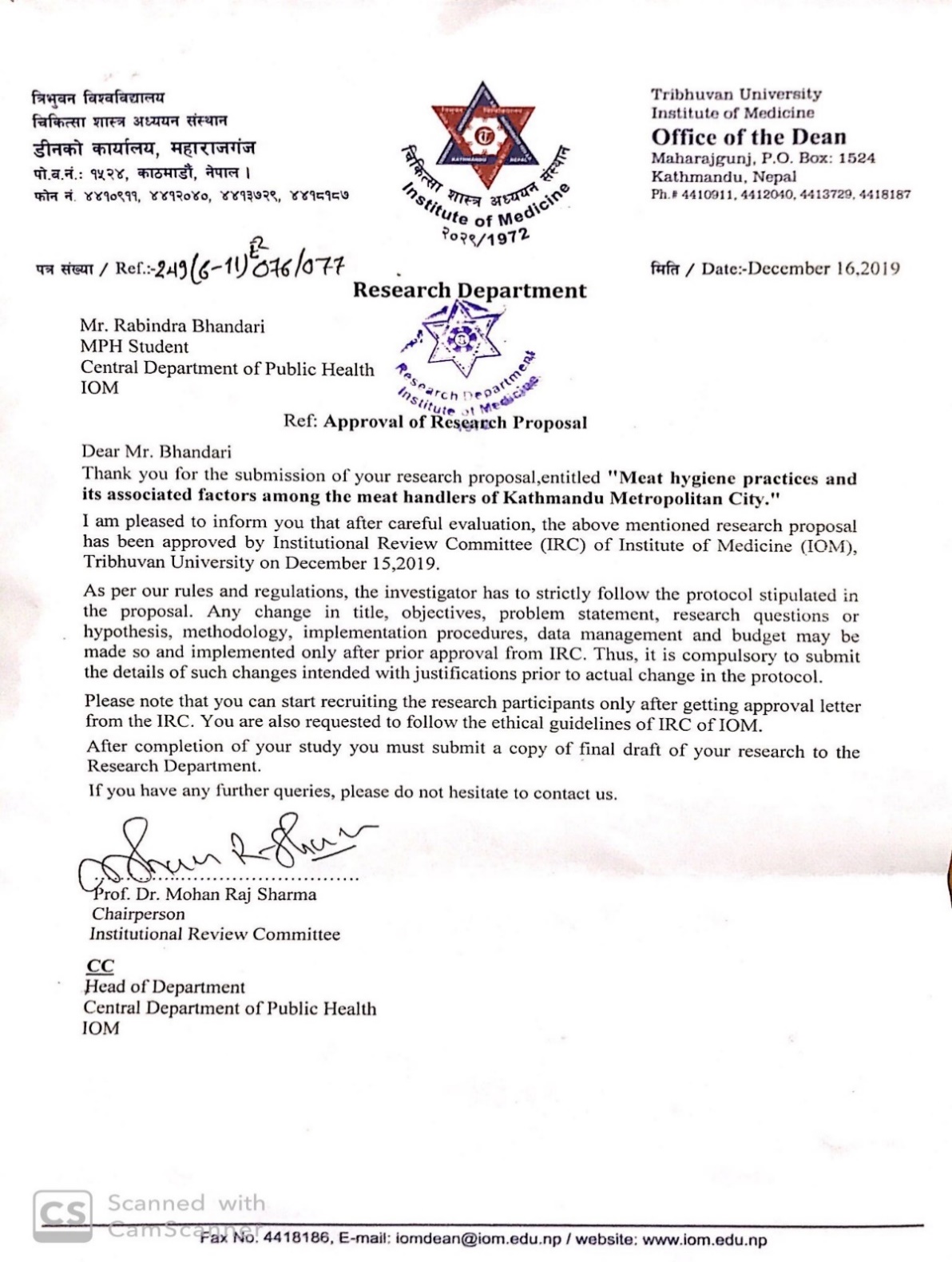

Supplement: S1 File — (DOCX) [file pgph.0001181.s002.docx]
