# Supplementary material for: Factors associated with meat hygiene-practices among meat-handlers in Metropolitan City of Kathmandu, Nepal
Source: PLOS Glob Public Health. 2022 Nov 9;2(11):e0001181. doi: 10.1371/journal.pgph.0001181 (PMC10021547; doi:10.1371/journal.pgph.0001181)
Supplement: S2 File — (DOCX) [file pgph.0001181.s003.docx]

## Survey Questionnaire in English

Code no: Ward no:

| **S.N.** | **Variables and questions** | | **Possible answers** | | **Remarks** | |
| --- | --- | --- | --- | --- | --- | --- |
| **A. Socio-demographic Variables** | | | | | | |
| **1.** | Age in completed years | |  | |  | |
| **2.** | 1. Sex: | | 1. Male 2. Female | |  | |
| **3.** | 1. Ethnicity | | 1. Brahmin 2. Chhetri  3. Janajati 4. Dalit  5. Madhesi 6. Muslim  7. Ethnic Minorities | |  | |
| **4.** | 1. Religion | | 1. Hindu 2.Buddhist  3. Islam 4. Kirant  5. Christian 6. …………. | |  | |
| **5.** | 1. Educational Status | | 1. No formal education  2. Primary level  3. Secondary level  4. Higher education | |  | |
| **6.** | 1. Major occupation of family | | 1. Agriculture  2. Meat selling  3. Government service  4. Foreign service  5. Business  6. Others (specify)……………….. | |  | |
| **B. Details of meat shop** | | | | | | |
| **1.** | 1. Location of shop | | 1. Market area  2. Residential area | |  | |
| **2.** | 1. Construction of shop | | 1. Open space  2.Temporarily constructed closed shop  3.Permanently constructed closed shop | |  | |
| **3.** | 1. Types of meat available | | …………………………………….. | |  | |
| **4.** | 1. How many workers are in the meat shop related to butchering? | | ……………………………………. | |  | |
| **5.** | Does your shop have license? | | 1. Yes 2. No | |  | |
| **6.** | 1. Do you have your own slaughtering place within the shop? | | 1. Yes 2. No | |  | |
| **7.** | If no from where do you bring slaughtered animals for meat? | | ………………………………………. | |  | |
| **8.** | How do you transport slaughtered animals/meat to the shop? | | 1. By carrying self  2. In cycle  3. Motorcycle  4. Car, Jeep or van  5. Others (specify) ……………….. | |  | |
| **9.** | What is the average transaction of meat per day? | | ……………………. In Kg. | |  | |
| **10.** | Are there facilities of temperature regulation for meat delivery (during transport)? | | 1. Yes 2. No | |  | |
| **11.** | In general how much time does it takes for meat to be sold which will be on display? | | …………………… in hours | |  | |
| **12.** | Are there facilities of cold storage (refrigerator/deep freeze) for long time storage? | | 1. Yes 2. No | |  | |
| **C. Detail of meat handlers** | | | | | | |
| **1.** | How long are you involved in meat handling? | | …………………… in years | |  | |
| **2.** | Do you have other job than butchering? | | 1. Yes 2. No | |  | |
| **3.** | From where have you learnt butchering? | | 1. Parents  2. Relatives/Friends/Self  3. Formal training | |  | |
| **4.** | What is your average workload per day? | | ........................Kg | |  | |
| **5.** | How long do you spend in slaughtering/cutting and selling of meat in a day? | | ........................Hours | |  | |
| **D**. **Awareness on meat hygiene among meat handlers** | | | | | | |
| 1. **1.** | Have you heard about hygienic meat? | | 1. Yes  2. No | |  | |
|  | If yes, What does it mean? | | 1. Fresh meat 2. Personal hygiene 3. Clean workplace 4. Fridging meat 5. Use of Chemicals 6. Others (specify)……………….. | |  | |
|  | Have you heard about unhygienic meat? | | 1. Yes 2. No | |  | |
|  | If yes, What does it mean? | | 1. Contaminated with dust, dirt 2. Contact with insects like housefly 3. Contact with rodents and waste from roadside 4. Contaminated with pathogens 5. Others (specify)……………….. | |  | |
|  | Have you ever heard about meat borne diseases? | | 1. Yes 2. No | |  | |
|  | What are the cause of meat borne diseases? | | 1. Chemicals  2. Germs  3. Supernatural power  4. Don’t know  5. Others ………………….. | |  | |
|  | What is the source of meat contamination?* | | 1. Infected animal  2. Infected slaughter house worker 3. Uncleansed equipments  4. Unmanaged and uncleansed setting  5. Accidental puncture of carcass’ GIT  6. Improper storage of meat  7. Don’t know | |  | |
|  | What are the health effects on humans eating unhygienic meat?* | | 1. Diarrhoea 2. Fever 3. Cough  4. Dermatitis 5. Musculo-skeletal problems 6. Malaria  7. Stomach pain 8.Repiratory difficulties 9. Skin rash 10. Tapeworm  11. Other (specify) ………………..  12. Don’t know | |  | |
|  | What are the hygienic aspects essential to be followed by meat handlers during and after slaughtering?* | | 1. Cleanliness of equipment  2. Cleanliness of setting  3. Personal hygiene  4. Protective clothing  5. Disposal of byproducts  6. Chilling of meat  7. Don’t know | |  | |
|  | What are the personal protective equipment essential for meat handlers?* | | 1. Aprons 2. Protective gloves  3. Foot wears 4. Masks  5. Hair cap | |  | |
|  | What is essential for cleanliness of equipment? | | 1. Water 2. Warm water 3. Soap and water 4. Soap and warm water  5. Disinfectant, soap and water 6. Disinfectant, soap and warm water | |  | |
|  | 1. How should the byproducts after slaughtering be disposed?* | | 1. Burial  2. Disposed in municipality waste 3. Supply to the fisheries  4. Food for pigs/dogs/cats or other animals  5. Others (specify) ………………..  6. Don’t know | |  | |
|  | Which diseases can affect meat handlers during the meat handling process? | | 1. Diarrhoea 2. Fever 3. Cough  4. Dermatitis 5. Musculo-skeletal problem 6. Malaria 7. Stomach pain 8.Repiratory difficulties 9. Skin rash  10. TB 11. Tapeworm  12. Other (specify) ………………..  13. Don’t know | |  | |
| **E. Meat hygiene practices** | | | | | | |
|  | | Do you keep the background information of the animal’s production place or disease that animal might have been affected with? | | 1. Yes 2. No | |  |
|  | | Do you have any system for inspection of animals or meat certification by the competent person or authority? | | 1. Yes 2. No | |  |
|  | | If yes at what interval? | | 1. Time to time 2. Every 3 months 3. Every 6 months 4. Once a year   5. Don’t know | |  |
|  | | What are the hygienic aspects essential to maintain meat hygiene while butchering? | | 1. Cleanliness of the equipment 2. Cleanliness of the setting 3. Personal hygiene 4. Meat hygiene 5. Disposal of byproducts 6. Others ……………… | | Multiple Responses |
|  | | Do you ever use PPE? | | 1. Yes 2. No | |  |
|  | | If yes, what are they? | | 1. Gloves 2. Cap  3. Mask 4.Apron 5. Boot 6.Goggles 7. Others …… | |  |
|  | | If no, what are the reasons for not using? | | 1. Not having appropriate knowledge  2. Not availability of PPE  3. Feeling uncomfortable  4. Inappropriate to use  5. Not having appropriate legal guideline for use | |  |
|  | | Do you use common equipment’s like knives, chop board, slab for processing meat? | | 1. Yes 2. No | | Ask only If the meat shop is selling 2 types of meat |
|  | | Do you clean the shop daily? | | 1. Yes 2. No | |  |
|  | | If yes, what do you use for cleaning? | | 1. Water only  2. Soap and water  3. Using chemicals (Surf, phenyl, etc.) | |  |
|  | | How often do you thoroughly clean the shop using detergent or disinfectant? | | 1. Once a month  2. Twice a month  3. Thrice a month  4. Weekly  5. More than once a week  6. Daily 7. Never | |  |
|  | | Is there provision of regular health examination or medical checkup of meat handlers? | | 1. Yes 2. No | |  |
|  | | Do you ever work during or when you are infect with some disease or illness? | | 1. Yes 2. No | |  |
|  | | Do you cover cuts or wound with waterproof bandage? | | 1. Yes 2. No | |  |

**E. Hygienic Practices (Observation Checklist)**

| **Main topic** | **Sub topic** | **Meat hygiene standards** | **Results ( Tick the observed practice)** |
| --- | --- | --- | --- |
| **Facilities and settings** | | | |
| Cleanliness of equipment | Knife | Clean and rust free | 1. Yes [ ] 2. No[ ] |
|  |  | Clean before each new period of work | 1. Yes [ ] 2. No[ ] |
|  |  | Sanitized before each new period of work or immersion in hot water in between the work | 1. Yes [ ] 2. No[ ] |
|  | Cutting board | Good state | 1. Yes [ ] 2. No[] |
|  |  | Clean before each new period of work | 1. Yes [ ] 2. No[ ] |
|  | Weighing machine | Clean or dirty | 1. Clean [ ] 2. Dirty [ ] |
|  | Utensils used | Clean or dirty | 1. Clean [ ] 2. Dirty [ ] |
|  |  | Sanitized before each new period of work or immersion in hot water in between the work | 1. Yes [ ] 2. No[ ] |
|  | |  |  |
| Cleanliness of setting | Bottom Floor | Impervious and Clean | 1. Yes [ ] 2. No[ ] |
|  | Walls | Impervious and clean | 1. Yes [ ] 2. No[ ] |
|  | Slab | Clean | 1. Yes [ ] 2. No[ ] |
|  | Ceiling | Clean | 1. Yes [ ] 2. No[ ] |
|  | Ventilation | Clean | 1. Available and clean air can pass 2. Not available  3. Available but improper |
|  | Separation within facility | Strict separation of clean and dirty areas | 1. Yes [ ] 2. No[ ] |
|  | Sewage | Good drainage | 1. Yes [ ] 2. No[ ] |
|  | Insects and animals | Insects/flies/animals | 1. Yes [ ] 2. No[ ] |
|  | Meat display | Contact with floor | 1. Yes [ ] 2. No[ ] |
|  | Lairage (cage)  (if present) | Clean | 1. Yes [ ] 2. No[ ] |
|  |  | Feed clean | 1. Yes [ ] 2. No[ ] |
|  |  | Clean drinking water | 1. Yes [ ] 2. No[ ] |
|  |  | Good drainage | 1. Yes [ ] 2. No[ ] |
|  | Disposal of byproducts | Frequent disposal | 1. Yes [ ] 2. No[ ] |
|  |  | Ways of disposal | 1. Burial [ ] 2.Disposed in municipality waste [ ] 3. Supply to the fisheries [ ] 4. Food for dogs /cats or other animals [ ] 5.Others (specify) [……………] |
| Other facilities | Storage | Refrigerator  If Yes, properly working  Cleaned regularly | 1. Yes [ ] 2. No[ ]  1. Yes [ ] 2. No[ ]  1. Yes [ ] 2. No[ ] |
|  | Water | Easily available warm water all the time | 1. Yes [ ] 2. No[ ] |
|  |  | Easily available water all the time | 1. Yes [ ] 2. No[ ] |
|  |  | Sink available for hand washing | 1. Yes [ ] 2. No[ |
|  | Lighting | Adequate | 1. Yes [ ] 2. No[ |
|  |  | If Yes, natural or artificial | 1. Natural [ ]  2. Artificial [ ] |
| **Materials present for Cleaning and sanitation** | | | |
|  | Soap | Washing body parts | 1. Yes [ ] 2. No[ ] |
|  | Disinfectant | Using for sanitation  Using for equipment’s | 1. Yes [ ] 2. No[ ]  1. Yes [ ] 2. No[ ] |
|  | Use of detergent | Using for cleaning | 1. Yes [ ] 2. No[ ] |
| **Meat handler’s health and behavior** | | | |
|  | Personal protective equipment’s | Apron | 1. Yes [ ] 2. No[ ]  If Yes,  1. Clean [ ] 2. Dirty [ ] |
|  |  | Gloves | 1. Yes [ ] 2. No[ ]  If Yes,  1. Clean [ ] 2. Dirty [ ] |
|  |  | Gumboot/shoe/slipper | 1. Yes [ ] 2. No[ ]  If Yes,  1. Clean [ ] 2. Dirty [ ] |
|  |  | Masks | 1. Yes [ ] 2. No[ ]  If Yes,  1. Clean [ ] 2. Dirty [ ] |
|  | Personal habits | Spitting in between | 1. Yes [ ] 2. No[ ] |
|  |  | Use of tobacco products in between | 1. Yes [ ] 2. No[ ] |
|  |  | Wiping hand with common clothes | 1. Yes [ ] 2. No[ ] |
|  |  | Counting money in between | 1. Yes [ ] 2. No[ ] |
|  |  | Doing other work in between without cleaning | 1. Yes [ ] 2. No[ ] |
|  |  | Nose picking | 1. Yes [ ] 2. No[ ] |
|  |  | Others | Specify……. |
|  | Cuts and Injuries (if any) | Covered with an appropriate water proof dressing | 1. Yes [ ] 2. No[ ] |
|  | Hand washing | Clean Hands | 1. Yes [ ] 2. No[ ] |
|  |  | Hand washing before, during and after work | 1. Yes [ ] 2. No[ ] |
